# Sympathetic Surgery.—I

**Published:** 1890-09-20

**Authors:** 


### Sympathetic Surgery—I.

There is a saying, shrewd and popular, that "the man who makes his own will has a fool for his lawyer." He may have the best intentions and the highest natural intelligence, but the lack of technical training in the work he undertakes will make him blunder egregiously in matters where a duller man who had undergone that training would succeed without trouble. One might supplement the saying we have quoted with another, to the effect that the man who attempts the cure of disease without being trained in the art of medicine has a fool for his physician. The "brilliant amateur" is a constant social danger, and one that becomes more alarming when he has once succeeded in his self-imposed task. He only knows that he has indeed succeeded, and gives all the credit to the treatment he has initiated, without regarding other factors which may possibly have influenced the event. When the Countess of Southdown administered "Daffy's Elixir" to a cottager's child undergoing the trying process of teething, and the little cherub got past the troublous time in safety, did her ladyship ever make allowance for the mother's care and the natural strength of the baby's constitution? Not at all. Daffy had done it; to Daffy be the glory. There is but one Daffy, and the Countess of Southdown is his prophetess. So the next ailing infant—a sickly creature, to whom opiates meant death—got a dose of Daffy, and succumbed to its soothing power. But that wasn't Daffy's fault. Certainly not. Blame the mother, the weather, the hapless babe itself, anything you like, except Daffy's Elixir and the Countess of Southdown.

When the doctors oppose this sort of treatment they are accused of "professional jealousy." There was a bad case of professional jealousy in the seventeenth century, when Sir Thomas Browne, M.D., of Norwich, laughed at Sir Kenelm Digby's great discovery of the cure of wounds by means of a sympathetic powder. This powder was applied, not to the wound itself, but to the weapon that wrought it, or to the bandage that first bound it up. The application of the preparation to these inanimate things at once cured the patient, even though he himself was miles away. This was the theory, simple and beautiful; and at it Sir Thomas laughed. Sir Thomas! The very name implies a sceptic mind. Knightly Sir Kenelm, who was not sceptical—indeed, we may infer from the trust he reposed his trust in his wife Venetia, a dame more fair than true, that he was rather credulous than otherwise—was huffed, in a stately way, at his opponent's contumacy; and though they argued it out by correspondence with the greatest courtesy, each left the other profoundly unconvinced.

The suffrages of an age like ours are almost certain to go with the doctor, but it would be rash to conclude that Sir Kenelm Digby was either a mere dreamer or an ignorant quack. He was a man of high scientific attainments, a student of chemistry and physical science, but he had no practical training in the art of surgery. He was a philosopher first and a physician afterwards, and it would have been better if the positions had been reversed. The strength and weakness of his intellect both are shown in the discourse he addressed to the learned College of Montpellier in defence of his pet nurseling, his Powder of Sympathy.

A copy of that oration lies before us now, a little volume bound in worn brown calf, and beautifully printed on paper which, though yellow with age, is stouter than that of many modern books. The elaborate title-page tells how and when the discourse was delivered, and bears as a motto the words, "*Felix qui potuit rerum cognoscere causas.*" Happy had Sir Kenelm been if he had sought the causes of things in less remote and fanciful theories! The discourse was taken down "in short writing upon the place as 'twas uttered," and translated out of French into English by R. White, Gent. We are further informed that the book is sold by Octavian Pulley, jun., at the Bible in St. Paul's Churchyard, over against the little north door; and the volume bears the date 1664.

The first thing that strikes us as we peruse the discourse is that the "Solemn Assembly of Nobles and Learned Men at Montpellier in France" who listened to it must have been dowered with no common stock of patience. Sir Kenelm has no idea of going straight to the point. He lays down principles—seven of them, with details and illustrations innumerable. The principles begin with the theory of light, its velocity and reflection, and end with the mild theorem that if you draw towards you a thread with a nail attached to one end of it you draw the nail too. But their bearing on the subject of the Sympathetic Powder is often either vague or illusory. As for the illustrations, they seem to be meant to show that Sir Kenelm has travelled far and wide and observed all he saw with an ingenious eye. He is too ingenious. His theories are sound enough, but having lived some two hundred years before Captain Cuttle, he had never heard the profound remark that "the bearings of this observation lays in the application on it." In "the application on it" he is mostly wrong. He lays too much on the wings of light. He is right in thinking that when the lines of light fall on some object which they cannot pierce] they rebound to some other; but it does not thence follow, as he imagines, that the light avenges itself on the obstinate body by carrying away loose particles of its substance.

Still, the theory is pretty enough, and not more fantastic than many newer ones. "The light beating," he says, "upon the body which opposeth her, cannot choose but make there some small incisions, proportionable to her rarity and subtility. And these small atoms being cut, and loosened from their trunk, being composed of the four elements (as all bodies are) the heat of the light doth stick, and incorporate itself with the most humid, viscous, and gluing parts of the said atoms, and brings them along with her." This theory of the attraction of atoms is, in fact, the foundation on which Sir Kenelm rests his belief in the Sympathetic Powder. The gory atoms on the sword or the bandage, being excited by the powder, are drawn to the similar atoms lying in the wound; once arrived there they fill their old spaces and the sore heals up. A finely simple explanation of a somewhat remarkable fact!

Let it not be supposed that we want to laugh at Sir Kenelm too much. He had grasped that discovery on which we moderns so pride ourselves, the germ theory of disease. He had realized that the air is no vacant ether, but is peopled almost to repletion with atomic emanations from every living thing. The eye could not see nor the touch feel them, but they were there, present and powerful. He had found out that the scent of the flower implied some infinitesimal loss of substance in the flower itself. He had guessed that the bloodhound on the track of a fugitive was guided by actual traces left on the ground, imperceptible as these were to human sense. Surely Sir Kenelm Digby was not far from Sir Joseph Lister.

But the application of the theory! There the enthusiast, the mystic, the man too keen to dwell on things undreamed of in our philosophy, comes in and overpowers the practical man. He uses his science to prop up superstitions and old wives' tales; he endows his atoms with conscious sentient power; he calls experiment to his aid, and summons "Euclide his Elements" to testify to something that never was in earth or sea or sky. The same spectacle has been witnessed since Sir Kenelm Digby's day.

For example—the knight is fond of examples, and wisely says that "in natural things we must have our last recourse to experience"—in the spring, when the vines are in blossom, the wines lying in cellars "begin to ferment, and put forth a little white lee upon the surface of the wine, which continueth in a kind of disorder until the flowers of the vine be fallen." What is the cause of this? The weather? That brief and British explanation would not have satisfied Sir Kenelm. He says it is the imprisoned wine-spirits in the cask striving after their free brethren in the vineyards. St. Ephrem the Syrian, our orator tells us, noted this in the third century after Christ, and observed also that dry onions bud in the house when those in the gardens begin to come out of the earth; "shewing thereby," he adds, "by these known examples of nature, the communication between living persons and the souls of the dead." Poor Saint Ephrem! neither he nor the honest knight who quoted him meant aught profane, and yet how grotesque seems the simile that proves our immortality by a string of onions!

*(To be continued.)*

